# Advances in Potential Cerebrospinal Fluid Biomarkers for Autoimmune Encephalitis: A Review

**DOI:** 10.3389/fneur.2022.746653

**Published:** 2022-07-22

**Authors:** Shuyu Zhang, Chengyuan Mao, Xinwei Li, Wang Miao, Junfang Teng

**Affiliations:** Department of Neurology, The First Affiliated Hospital of Zhengzhou University, Zhengzhou University, Zhengzhou, China

**Keywords:** Autoimmune encephalitis (AE), biomarker, cerebrospinal fluid, cytokines/chemokines, demyelination, nerve damage

## Abstract

Autoimmune encephalitis (AE) is a severe inflammatory disease of the brain. Patients with AE demonstrate amnesia, seizures, and psychosis. Recent studies have identified numerous associated autoantibodies (e.g., against NMDA receptors (NMDARs), LGI1, etc.) involved in the pathogenesis of AE, and the levels of diagnosis and treatment are thus improved dramatically. However, there are drawbacks of clinical diagnosis and treatment based solely on antibody levels, and thus the application of additional biomarkers is urgently needed. Considering the important role of immune mechanisms in AE development, we summarize the relevant research progress in identifying cerebrospinal fluid (CSF) biomarkers with a focus on cytokines/chemokines, demyelination, and nerve damage.

## Introduction

Autoimmune encephalitis (AE) refers to a type of encephalitis caused by an autoimmune inflammatory response, which is characterized by abnormal mental behavior, epilepsy, and memory impairment. Different antibodies may have different clinical manifestations. Early diagnosis of AE is difficult due to atypical clinical symptoms and inconclusive laboratory examination results. So far, AE patients suffer from limited treatment methods and long-lasting treatment cycle, and those who are severely affected also faced with higher treatment cost and mortality. They may also sustain relapse that seriously affects the life quality and causes huge social burden.

**Figure 1:**
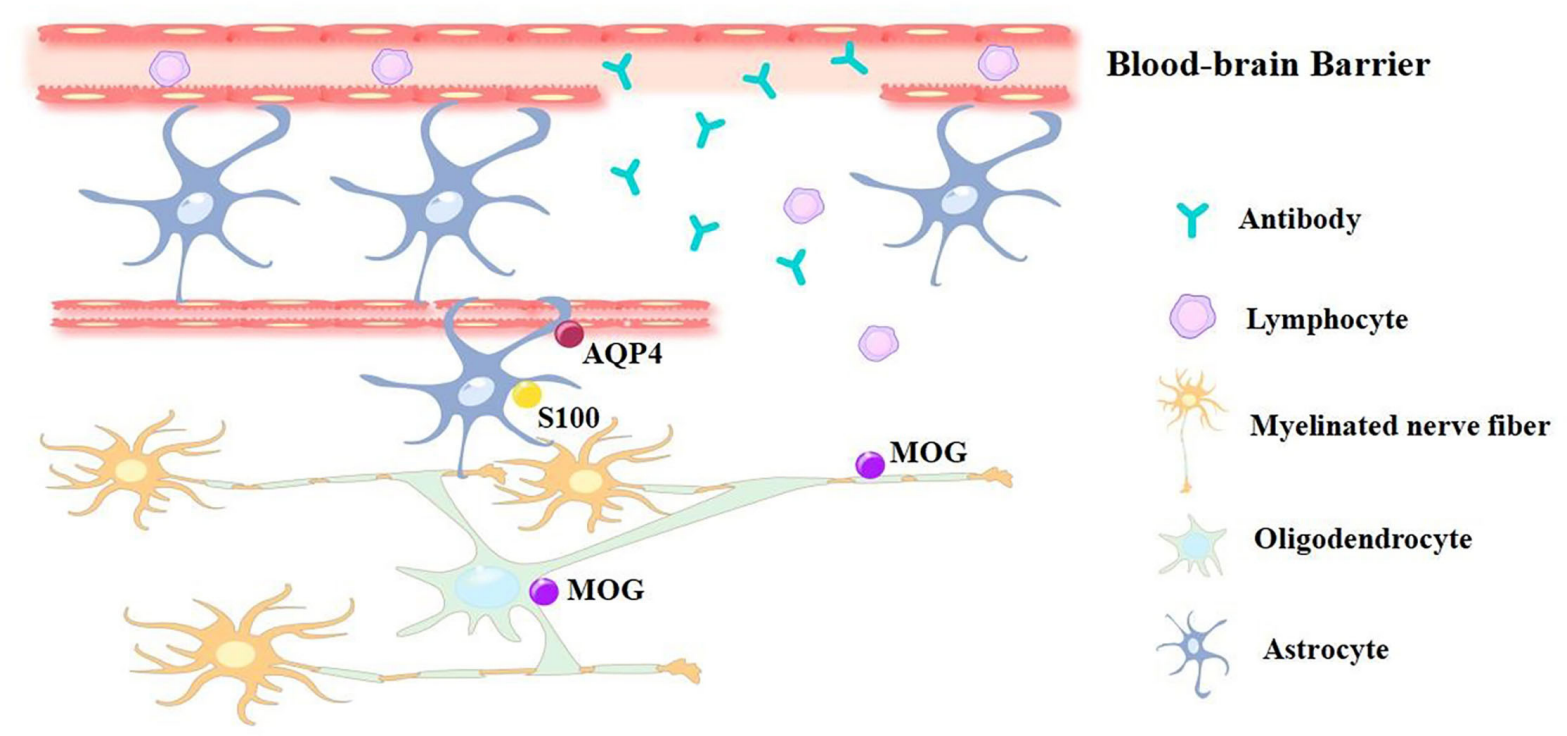
The pathological effect caused by blood-brain barrier damage. The blood-brain barrier damage during AE progress is caused by Th17 cell activation, this leads to antibodies and lymphocytes entering the brain and cerebra-spinal fluid. Traditional methods for AE diagnosis are using CFS to detect relevant positive Ab, while novel approach to diagnose AE are using potential biomarkers, including cytokines/chemokines and molecules like MOG, AQP4 and S100 protein that expressed on nerve cells.

As the most prevalent classification method, Graus et al. (1) sorted AE based on antibodies against neuronal cell-surface or synaptic proteins. These antibodies are known as antibodies against intracellular antigens, antibodies against synaptic receptors, antibodies against ion channels, and other cell-surface proteins, respectively. Since the discovery of anti-N-methyl-D-aspartate receptor (NMDAR) encephalitis in 2007, many autoimmune antibodies related to AE have been discovered, leading to a sharp increase in the detection rate compared to that of infectious encephalitis (2–4). The relevant autoantibodies are mainly directed against NMDAR, leucine-rich glioma-inactivated 1 (LGI1), γ-aminobutyric acid A / B receptors (GABAA/B), contact in-associated protein 2 (CASPR2), α-amino-3-hydroxy-5-methyl-4-isoxazolepropionic acid receptor (AMPAR), dipeptidyl-peptidase-like protein-6 (DPPX), and voltage-gated potassium channel (VGKC). Among them, anti-NMDAR encephalitis is the most common and easily recognized form of AE (5–12). How these pathological autoantibodies enter the brain to cause neuropathology and affect neural circuits remains unclear (13). Studies show that viral infection, tumors, and other factors can result in AE (14). Molecular stimulation or induction of antigen release is the most important pathogenesis, leading to the production of autoantibodies. Once these antibodies recognize neuronal receptors or synaptic proteins as foreign epitopes, the antibody-mediated AE is triggered (13, 15, 16). Changes in CD4^+^ and CD8^+^ T-cells in peripheral blood and CSF has also been reported using flow cytometry, especially in autoimmune limbic encephalitis (17–20). The diagnosis of AE is difficult during the early stages due to the varied symptoms and atypical results in routine cerebrospinal fluid (CSF) examination (21, 22). Currently, the diagnosis of AE depends on positive antibody detection; however, the absence of antibodies does not exclude the possibility of disease, and the measurement of the antibody level is also difficult to achieve. It is unrealistic to use the antibody detection as a necessary condition for early diagnosis (23, 24). Moreover, since live cells are not conventionally available, antibodies are generally investigated with fixed cells during routine clinical practice, which makes it easy to obtain false results. The antibody levels cannot predict disease prognosis and recurrence as well (1, 5, 25–27). Therefore, it is critical to discover novel inflammatory CNS biomarkers, especially CSF-specific markers, for clinical applications (Figure 1).

Recent studies have confirmed that both humoral and cellular immunity are involved in the pathogenesis of AE (9, 28–35). In addition to the production of antibodies that can target corresponding nerve cells, neuroimmune-regulatory mechanisms also play an important role in disease progression. B and T lymphocytes are the core of humoral and cellular immunity, respectively, and their differentiation, development, and migration largely depend on cytokines and chemokines. Besides, immune-related demyelination of the nervous system, nerve cell damage, and genetic susceptibility are closely related to the occurrence of autoimmune diseases. This review provides a brief summary of this research in identifying autoimmune CSF markers and related treatments based on the factors mentioned earlier.

## Cytokines/Chemokines

**Figure 2:**
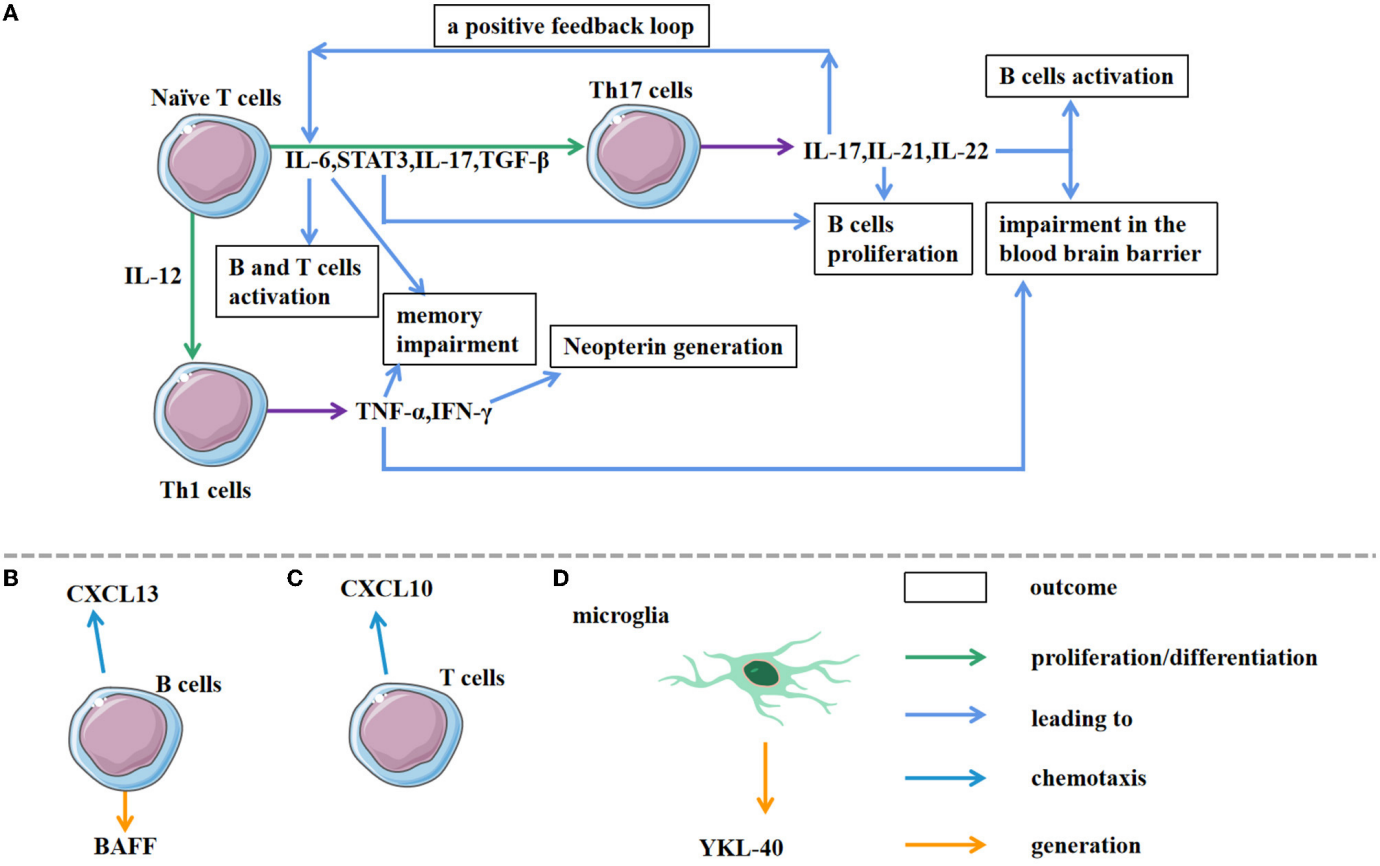
Associated cells involved in AE pathological processes. **(A)** Under the action of different cytokines, naive T cells differentiate into different Th cells. As a joint result of IL-6, STAT3, IL-17, TGF-β, naive T cells differentiate into Th 17 cells. The subsequent production of IL-17, IL-21, IL-22 by Th 17 cells triggers the immune response and impairs the blood brain barrier. In addition, IL-17A triggers a positive feedback loop of IL-6 signaling, leading to IL-17A/IL-6 co-activation. IL-12 activation can induce Th 1 cell differentiation. Furthermore, Th 1 cells secrete cytokines, such as TNF-α and IFN-γ, resulting in different outcomes. **(B,C)** As chemokines, CXCL13 and CXCL10 induce directional chemotaxis of B cells and T cells, respectively, towards the target sites. **(D)** The production of YK-40 by microglia contributes to the early diagnosis.

Cytokines are associated with the pathogenesis of a variety of autoimmune diseases (36, 37). They are important immunoregulatory factors that participate in both humoral immune responses such as B cell recruitment and differentiation, plasma cell maturation, and antibody secretion, and in cellular immune responses such as T cell differentiation and cytotoxic cell function. Chemokines are small cytokines or signal proteins secreted by various types of cells, which induce directional chemotaxis of nearby immune cells. When the body defends and clears invading pathogens, chemokines direct immune cells toward the sites of infection. Cytokines/chemokines exert multiple effects on many inflammatory cells (Figure 2A), and most of them possess unique characteristics and are elevated in many neuroinflammatory diseases of the CNS, indicating that they may be potential biomarkers (36). They are also important in disrupting the normal blood-brain barrier (BBB) and the subsequent B cell infiltration (13, 38–40). Studies have shown that cytokines and chemokines can be used as biomarkers for the diagnosis of autoimmune diseases and the detection of intrathecal inflammation. As quantifiable indicators, cytokines have the potential to be used as novel biomarkers for the evaluation of AE (41), with the cytokine profile differing depending on the antibody subtype (30). Interleukin-6 (IL-6), IL-17A, C-X-C motif chemokine ligand 10 (CXCL10), and CXCL13 in CSF have been reported to be useful for inflammation detection during the acute phase of AE while IL-15 or chitinase-3-like 1 (CHI3L1) may exhibit chronic disease activity (42). Clinical analyses of chemokine and cytokine levels would help to better understand the immune process of this disease.

### Factors That Promote the Differentiation and Development of Th17: IL-6, Transforming Growth Factor-β, and Signal Transducer and Activator of Transcription 3 (STAT3)

Impairments in cytokine/chemokine regulation are associated with the occurrence of AE. A large amount of cytokines and chemokines participate in the chemotaxis, differentiation, and development of Th17 cells, which have great significance in the occurrence and progression of a variety of autoimmune diseases (43, 44). Activation of excessive Th17 cells can lead to inflammation and demyelinating diseases of the CNS (45–47). As an inflammatory Th subset, Th17 cells involve in intrathecal synthesis and B cell activation. A previous study has demonstrated Th17 cell accumulation in the CNS of AE patients by comparing the CSF of 60 randomly selected anti-NMDAR encephalitis patients to patients with non-inflammatory diseases as negative control using Fluorescence Activating Cell Sorter (FACS) analysis (28). Animal experiments have shown that Th17 cells can degrade the BBB, allowing lymphocytes, antibodies, and other substances to enter the CNS. The RORγt^−/−^ Th17 deficient mouse model also manifested reduced regulatory T cell numbers and attenuated microglial activation during post infectious basal ganglia encephalitis (BGE), a subset of AE syndromes (13). These findings highlight the key role of Th17 cells during AE activation. Cytokines/chemokines such as IL-6, IL-17 and TGF-β as well as STAT3 are the main factors that can promote the differentiation of CD4+ T cells to Th17 cells (28, 30, 43, 48, 49). IL-6 is a pleiotropic cytokine of great concern in regulating the immune system. As a powerful pro-inflammatory cytokine, it is vital for hosting resistance to pathogens and acute stress. Moreover, IL-6 counts a great deal in the auto antibody production and T cell activation. Therefore, it heavily participates in the inflammatory cascade involving T and B cell (49, 50). STAT3 acts as a carrier during interactions between cytokines and their receptors, which is mainly responsible for maintaining cellular signal transduction. It has been shown to be an important regulator of many anti-apoptotic genes. TGF-β can be produced by a variety of cells to regulate cell growth and differentiation.

Studies have reported that the pro-inflammatory cytokine IL-6 is elevated in the CSF of patients with anti-NMDAR encephalitis (30, 37, 51), but this increase is not remarkable in patients with anti-LGI1 encephalitis (30). IL-6 can attenuate the effects of NMDA-mediated excitatory postsynaptic currents (EPSCs), which contribute to memory impairment of AE patients (52). It can also strongly induce Th17 cell differentiation *in vitro* and may act independently to promote Th17 development and cause related autoimmunity *in vivo* (53). TGF-β and STAT3 activation can induce Th17 cell differentiation and participate in immune responses as well. These cytokine/chemokine-induced Th17 cell changes and immune reactions contribute to the occurrence of AE (28).

### Cytokines Secreted by Th17cells: IL-17, IL-21, and Tumor Necrosis Factor-α (TNF-α)

Th17 cells promote tissue inflammation by inducing the production of pro-inflammatory cytokines, including IL-17, IL-21, and TNF-α (28, 41, 43, 47, 54–56). As these factors are all products of Th17-immunity which has been reported to be activated in AE, it is likely that they can be candidate AE biomarkers.

IL-17 secreted by Th17 is an early initiation factor of the inflammatory response, considering effects *via* recruiting granulocytes and macrophages (43, 57). Anti-NMDAR AE patients are usually accompanied by the IL-17 increase in their CSF and serum (30, 33, 44). IL-17 can disrupt the function of tight junctions and promote the passage of inflammatory cells through BBB. It can also promote the secretion of cytokines in a variety of cells, which participate in cell differentiation and development and cause inflammation (58–61). IL-17A may trigger a positive feedback loop of IL-6 signaling through NF-κB and STAT3, leading to the development of autoimmune diseases (62). Thus, IL-17A/IL-6 co-activation in anti-NMDAR encephalitis may be a key factor in the pathogenesis of the disease and the production of intrathecal antibodies (30).

Autocrine regulation causes Th17 cells to secrete IL-21, and this can in turn promote B cell proliferation and differentiate into plasma cells. IL-21 downregulates the functions of FOXP3+ regulatory T cells to enhance autoimmunity (63–65). Jiang et al. collected 32 AE patients, 5 patients with other autoimmune neurological disease, and 10 patients with non-inflammatory disease. A study has found a significantly increased IL-21 level in AE patients compared to control groups and even the detected levels of IL-21 in the CSF were in the low range of the assay (0–6 pg/ml). It is proposed that IL-21 may be a promising surrogate biomarker for the diagnosis of AE (41).

TNF-α is increased in the CSF of patients with anti-NMDAR encephalitis. It can reduce the integrity of the BBB and can be used to diagnose and monitor intrathecal inflammation. Recent studies have confirmed that the appearance of cognitive impairment in patients with AE is associated with TNF-α (51, 52, 66). A localized increase of TNF-α in the hippocampal dentate gyrus activates astrocyte TNF receptor type 1 (TNFR1), which then triggers astrocyte-neuron signaling cascades, leading to sustained functional changes in hippocampal excitatory synapses (66). This illustrates that during pathological conditions including Alzheimer's disease (AD), TNF-α is harmful for memory function and synaptic plasticity, and TNF-α inhibition can be used to effectively manage the disease (67, 68).

### Immune-Related Chemokines: CXCL10, CXCL13, C-C Motif Ligand 19, CCL20, and CCL22

Chemokines attracted immune cells to the sites of inflammation and could be responsible for the initial pleocytosis (33). This makes them important in exploring the function of Th17 cells in patients with anti-NMDAR encephalitis (28). CCL19, CCL20, CCL22, CXCL10, CXCL13, and other chemokines are elevated in the CSF of patients with anti-NMDAR encephalitis. Some of these chemokines can increase in CFS cells and result in the early progression of the disease (28). Therefore, chemokines may be potential biomarkers for the diagnosis and monitoring of intrathecal inflammation.

CXCL13 is the main determinant of B cell recruitment during CSF neuro inflammation (Figure 2B) (69). The increased concentration of CSF CXCL13 in patients with anti-NMDAR encephalitis is associated with the synthesis of intrathecal anti-NMDAR antibodies, and thus, it may be a possible biomarker for evaluating the treatment response and prognosis (33, 70). CXCL13 is also the key chemokine that recruits plasma cells; the levels of CXCL13 in CSF are correlated with the abundance of CSF plasma cells or plasmablasts (30, 71, 72). Notably, elevated CXCL13 levels in CSF are specific to anti-NMDAR encephalitis instead of anti-LGI1 encephalitis, which may be due to the different IgG subtypes between the two diseases (30). The persistence or secondary increase of CXCL13 is related to the recurrence of the disease and the limited response to treatment. Second-line treatment is recommended if the CXCL13 level remains high after first-line immunotherapy (70). Studies have suggested that CXCL13 and CXCL10 (a chemokine for T cells) are both elevated in the early-stage anti-NMDAR encephalitis (approximately 40 days), and they are associated with the complexity and early progression of the disease. As the disease develops (approximately half a year), CXCL13 gradually decreases while CXCL10 remains elevated (33), supporting the hypothesis that the B cells take part in abnormal inflammatory activation in the early stages of the disease whereas T cells participate in immune regulation. The appearance of CD4+ T cells in the lesion is induced by CXCL10 (Figure 2C). Therefore, some central chemokines such as CXCL13 and CXCL10, may be latent therapeutic targets for AE. Additionally, cytokines/chemokines like migration inhibitory factor (MIF), CCL2, CCL20, and CCL22 that act as monocytes/macrophages may continue to increase in the CSF of patients with anti-NMDAR encephalitis for several months during the recovery period. For patients with residual behavioral abnormalities, MIF and CCL2 have been reported to persist for 256 days (72). Relative studies also demonstrated that anti-NMDAR patients have a higher concentration of CCL20 and CCL22 using the ELISA assay (28). B cell activating factor (BAFF) is a pivotal indicator of B cell activation and survival, but it has been reported that there is no significant increase in BAFF in the CSF of anti-NMDAR encephalitis patients. The specific role of certain chemokines requires further study.

### Other Factors Involved in Immunity: Neopterin, CHI3L1, and Osteopontin (OPN)

Neopterin is a purine-like molecule released by monocytes and macrophages, which is activated by γ-interferon. It is a marker of cellular immune activation and can induce the expression of pro-inflammatory NF-κB, intercellular adhesion molecule-1, cytokines, and other inflammatory mediators (73–75). Neopterin production is closely related to infection, especially viral infection, and the increasing level of neopterin can be seen in various inflammatory and autoimmune CNS diseases. Researchers have found that neopterin is involved in B lymphocyte proliferation, meanwhile, autoantibody production by B lymphocytes and plasma cell infiltrates is found in the CNS of AE patients. As AE is closely linked to autoimmunity and inflammation, neopterin can be a novel biomarker for AE (25, 76). It is noted that, as neopterin has a short half-life, it can be used to monitor the inflammatory activity in patients with acute inflammation or recurrent encephalitis. It is of great importance as early diagnosis of AE depending on existing Ab tests is not realistic.

CHI3L1 (YKL-40) is mainly expressed in microglia, especially during acute and chronic inflammatory reactions (Figure 2D) (77, 78), so, it is considered to be a probable marker of persistent inflammation in many diseases (79–81). As mentioned above, Il-6 participates in Th17-immunity, which is crucial for AE development. IL-6 signaling can stimulate B cell proliferation and up-regulate the expression of the inflammatory marker CHI3L1 (82). An increase in CHI3L1 levels in the brain and/or CSF is seen in various immune-related diseases, especially neurological and neurodegenerative disorders with inflammatory components like multiple sclerosis (MS), AD, and stroke (83–85). Interestingly, the CHI3L1 levels are elevated in the CSF of patients with anti-NMDAR encephalitis and correlated with the clinical MRS score. This suggests that CHI3L1 may be a biomarker for the prognosis of anti-NMDAR encephalitis (51, 82).

OPN is a phosphorylated protein expressed in various cells (86), which is vital in many pathophysiological processes including inflammation and immune responses (87, 88). OPN can induce B cell proliferation and antibody production, and it is also crucial in Th17 cell differentiation. As mentioned earlier, Th17 cells partake in intrathecal antibody synthesis and B cell activation, hence contributing to anti-NMDAR encephalitis (89–91). Therefore, OPN may also be used as a biomarker of anti-NMDAR encephalitis (82).

## Nerve Damage and Glial Activity Markers

The occurrence of CNS diseases including AE may lead to nerve damage. For example, by actively participating in AE progression, inflammatory events can directly cause the damage of nerve cells including neurons and oligodendrocytes (92, 93). Patients with AE often present with leukoencephalopathy syndrome (94–97), which occurs simultaneously or in succession (98). As structural elements of neurons and glial cells, the neurofilament light (Nfl), total tau protein (T-tau), and glial fibrillary acidic protein (GFAP) imply neuronal and glial cell damage and death. Their elevation has also been found in the CSF of patients with AE (99–101). Therefore, these markers may indicate abnormal intracranial damage in patients with AE and accordingly guide clinical diagnosis and treatment.

### S100 Protein

The S100 protein belongs to the calcium-binding protein family and has two important members, the related homo-dimeric proteins S100A and S100B. S100B is a CNS-specific protein, mainly produced by astrocytes. It is a biochemical marker for brain injury, and the S100B concentration is closely related to the injury degree, treatment efficacy, and prognosis (102, 103). Many factors such as TNF-α can stimulate the release of S100B (104). Studies have manifested that the concentration of S100B in the CSF of patients with anti-NMDAR encephalitis is significantly higher than that in the control group. Additionally, there is a strong correlation between the concentration of S100B in CSF and the MRS score of the patient, suggesting that CSF S100B is related to the prognosis of the disease (105). S100 protein is also detectable in the CSF of patients with anti-DPPX encephalitis (106). Other studies have shown, however, that the concentration of S100B is AE-independent (107). S100A6, on the other hand, belongs to the S100A protein family and can help B lymphocytes to pass through the BBB in AE patients (108).

### Progranulin (PGRN)

PGRN is a multifunctional immunomodulatory molecule which is critical in autoimmune diseases such as systemic lupus erythematosus and vasculitis (109). It has a double effect of inflammation, which not only can promote inflammation by regulating the IL-6 signaling pathway, but also can have an anti-inflammatory effect by antagonizing the TNF-α signaling pathway or regulating IL-10 and other signaling pathways (110–112). PGRN can promote the differentiation of CD4+ T cells into regulatory T cells (Tregs) and enhance their functions (111). The level of PGRN is elevated in the CSF of patients with anti-NMDAR encephalitis, indicating that the concentration of PGRN in the CSF may be a prospective biomarker of acute anti-NMDAR encephalitis. In patients with anti-LGI1 encephalitis, on the contrary, the PGRN in the CSF remains normal. Although PGRN is also considered to be a biomarker for certain tumors (such as lymphoma) as well, PGRN is not increased in the serum or CSF of AE patients with paraneoplastic syndrome (113, 114).

### Neurofilament Light (Nfl)

Neurofilament light (NfL) chains are scaffolding proteins expressing specifically on the neural skeleton. They have been used as unspecific markers of axonal damage during neurodegeneration and neuroinflammation. To investigate the association between the CSF-Nfl level and AE, researchers collected CSF from AE patients including 37 of anti-NMDAR encephalitis and 16 of anti-LGI1 encephalitis. They found that compared to the control groups and AE patients without Nfl, people who have either of these 2 types of AE accompanied by CSF-Nfl elevation manifested poor diagnosis and prognosis. There is also a strong correlation between CSF-Nfl and AE pathogenesis (115). Moreover, there is also a significant difference among anti-NMDAR AE patients with various etiologies (114, 116). Kammeyer et al. (117) collected 26 plasma and 14 CSF samples of patients with autoimmune neurological disorders, compared to the control group, and patients with active AE show an elevated level of plasma Nfl, implying that it may act as a minimally-invasive biomarker for CNS injury during AE development. Another study also confirmed the opinion that the Nfl level is closely related to disease severity during diagnosis (118).

### GFAP

GFAP is a key component of the cytoskeleton during astrocyte development, and it is also an essential filament protein in mature astrocytes (119). As neuronal and glial cell damage markers in CSF, GFAP increased non-specifically in patients with brain disorders (120). During brain injury, full-length GFAP from injured astrocytes entered the subarachnoid CSF, which then circulated to the peripheral blood through direct venous drainage. Researchers gathered clinical data from 25 AE patients, and the follow-up analysis found that the GFAP levels in the CSF of AE patients were moderately elevated during the early stage of disease development. The final outcome (disability at 1 year) strait was associated with the CSF-GFAP levels at all time points (101).

### T-Tau

As a representative marker of neuronal and axonal loss, T-tau has long been used for the diagnosis of neurodegeneration. A recent study has revealed a direct connection between AE and atypical tauopathy (121). By comparing the serum and CSF level of T-tau along with MRI data from 13 AE patients and their age-matched controls, Peter Körtvelyessy et al. found that patients with the temporal FLAIR-signal in the MRI and those developing hippocampal sclerosis are prone to have a highly increased T-tau level in CSF (114). Another research collected relevant data from 25 patients hospitalized for AE and followed for 1 year, and a high CSF level of T-tau was found during the acute phase of AE. The final outcome is directly linked to the CSF-T-tau level at around 3 months since the disease onset (101).

## Oligoclonal Bands (OCBs)

OCBs are clones of immunoglobulins and can be detected in CSF or serum (122). They are related to several diseases mediated by many factors such as auto antibodies, demyelination, infection, and inflammation. Patients with anti-NMDAR encephalitis and other rarer subtypes like anti-GABABR, anti-AMPAR, and anti-DPPX encephalitis, can show OCB positive in CSF and /or serum, but patients with anti-LGI1, anti-IgLON5, anti-CASPR2, and anti-GlyR antibodies are rarely OCB positive (5, 6, 123–128). Studies have shown that the levels of OCB are correlated with an increase in the CSF cells in AE patients (5). Autoantibody negative encephalitis is a type of AE with seronegative CSF, which caused the antibody testing as non-pathognomonic. The CSF OCB positivity rate is included in the clinical diagnostic criteria for both anti-NMDAR encephalitis and possible AE with negative antibodies (1). The presence of OCBs is related to the late manifestations of anti-NMDAR encephalitis in patients with decreased consciousness, movement disorders, and autonomic symptoms (129). Moreover, OCBs predict the presence of cancer at the time of AE diagnosis. In patients with OCB-positive CSF, strict monitoring of potential malignant tumors should be considered even before knowing the results of the neuro-autoantibody tests (130).

In pediatric patients with anti-NMDAR encephalitis, mirror OCBs develop initially and then progress into intrathecal OCBs, suggesting that these patients develop a systemic autoimmune response in the beginning, which is followed by localized intrathecal antibody synthesis (122). Compared with intrathecal OCBs, mirror OCBs have a lower specificity for CNS inflammation. Mirror OCBs indicate the presence of OCBs in serum and CSF. Recent studies have demonstrated that autoimmune-related CNS disease antibodies are produced in the peripheral circulation (131, 132). Therefore, mirror OCBs should not be ignored, as they may be an important biomarker of inflammatory CNS diseases.

## Markers of Synaptic Dysfunction

The increase of synaptic-associated proteins is reported in the CSF of patients with neurodegenerative diseases, suggesting an abnormal synaptic integrity and function during disease pathogenesis. Among them, neurogranin and synaptosomal-associated protein-25 (SNAP-25) are the 2 representative pre- and post-synaptic proteins, respectively (133, 134). To clarify whether these markers can also reflect the progression of antibody-mediated encephalitis (AME), researchers obtained CSF from the diagnosis of 45 patients as AME including the NMDA receptor (*n* = 34) and LGI1/CASPR-2 (*n* = 11). By comparing with 39 age- and sex-similar health control, both neurogranin and SNAP-25 were markedly decreased in the CSF of AME patients at presentation. Lower SNAP-25 in prospectively followed patients and higher neurogranin at presentation are associated with greater disease severity (135). The decreased synaptic protein level is probably due to acute synaptic dysfunction and antibody-mediated receptor internalization, which may correlate with disease severity and outcome (135).

## Prospects for Treatment

The most common treatment strategies for AE are the first-line treatments such as corticosteroids, IVI g, and plasma exchange as well as the second-line treatments like rituximab, cyclophosphamide, mycophenolate mofetil, and other immunosuppressive medication (38, 136–140). It has been reported that 44% of patients with anti-NMDAR encephalitis have failed first-line treatment, demonstrating that individual patients may have specific immunological characteristics. Compared with non-targeted immunotherapy, targeted immunotherapy has the potential to improve the treatment response, and further research on targeted immunotherapy will be necessary to develop new treatment strategies (141).

Th17 cells are important immune cells involved in the pathogenesis of AE. Inhibiting the differentiation and development of Th17 cells can inhibit the development and progression of inflammation. Studies have shown that the cAMP-response element binding protein (CREB)-regulated transcription coactivator (CRTC2) can promote the differentiation of Th17 cells, therefore, its inhibitors may provide therapeutic benefits for patients with autoimmune diseases. Although there are no reports on inhibiting Th17 cells to treat AE currently, this may be a viable therapeutic strategy for AE in the near future (43). Additionally, IL-2 can constrain the differentiation of Th17 cells, and low-dose IL-2 therapy can be used to treat autoimmune diseases by restoring the balance between Tregs and effector T cells (30, 142). The low-dose IL-2 usage may be a feasible immunotherapy for refractory AE treatment.

Cytokines play a critical role in the autoimmune response. Some cytokine inhibitors, such as anti-IL-6 receptor antibody (tocilizumab), IL-17A inhibitor (secukinumab), and CXCL-10 inhibitor (50, 52, 143, 144) have been shown to block CNS immune feedback pathways and antigen-specific Th17 cell differentiation (145), and they can also effectively treat many autoimmune inflammatory diseases. AE-related cytokine inhibitors and additional cytokines/chemokines involved in AE may be identified as potential targets for future treatments (44, 146–148).

Furthermore, research on NR2B antagonists may yield potential therapeutic options (149) to protect the BBB and exhibit effective therapeutic effects without requiring antigen specificity (150).

## Conclusion

Recently, AE has received increasing attention, and the level of AE diagnosis and treatment has dramatically improved. However, there are drawbacks for clinical diagnosis based solely on antibodies; therefore, additional biomarkers are needed to guide diagnosis and treatment. Considering the primary role of the immune mechanism in the pathogenesis of AE, this review summarizes the relevant research progress in identifying CSF biomarkers with a focus on cytokines/chemokines, demyelination, and nerve damage (Table 1). Furthermore, we also provide the latest information to aid the diagnosis and treatment of the disease. Additional research will increase our understanding of AE and improve the level of diagnosis and treatment for this disease.

**Table 1:** Concise summary of potential AE biomarkers.

**Marker types**	**Sub-types**	**Published putative function in AE pathogenesis**	**Potential use (diagnosis, prognosis, treatment response, etc)**	**Changes in specific AE subtype**	**Degree of importance**	**References**
Cytokine and chemokines	Factors that promote the differentiation and development of Th17 cells: IL-6, TGF-β, and STAT3	IL-6, TGF-β and STAT3 are all upstream signal molecules of Th17 cells. Combined with TGF-β, IL-6 can promoteTH17 cell differentiation mediated by STAT3. Th17-immunity has been reported to be activated in AE	Factors mentioned in this part are mainly Th17 cell-associated. Th17 cells accumulation is correlated with poor prognosis of AE, especially anti-NMDAR AE. CXCK13 may be a potential marker of treatment response and relapse rate. Meanwhile, as indicators for B cell and T cell respectively, CXCL13 and CXCL10 can reflect the disease process as B cells take part in abnormal inflammatory activation in the early stages of the disease whereas T cells participate in immune regulation Neopterin can reflect the acute and recurrent encephalitis during diagnosis.	IL-6: ↑ in anti-NMDAR encephalitis	★★★	(28, 50)
	Cytokines secreted by Th17 cells: IL-17, IL-21, and TNF-α	IL-17, IL-21 and TNF-α downstream signal molecule of Th17 cells. Produced by Th17 cells during inflammation. Th17-immunity has been reported to be activated in AE.		IL-17: ↑ in anti-NMDAR encephalitis TNF-α: ↑ in anti-NMDAR encephalitis	★★★	(28, 41, 43, 47, 52, 54, 70)
	Immune-related chemokines: CXCL10, CXCL13, CCL19, CCL20, and CCL22	CXCL13 and CXCL10 are responsible for B cell activation and T cell chemotaxis respectively, which are all reported to be activated in AE. CCL20 and CCL22 promote Th17 cells migration. Th17-immunity has been reported to be activated in AE.		CXCL13: ↑in anti-NMDAR encephalitis CCL20: ↑in anti-NMDAR encephalitis CCL22: ↑in anti-NMDAR encephalitis	★★★	(28, 70)
	Other factors involved in immunity: Neopterin, CHI3L1, and OPN	Neopterin is a marker for cell immunity activation and can induce many inflammatory mediators, which reported to participate in AE progression. CHI3L1 is mainly expressed in microglia during inflammation, which participate in AE pathogenesis. OPN can induce B cell proliferation and antibody production, it is also crucial in Th17 cell differentiation, hence contributing to the development of AE.		CHI3L1: ↑ in anti-NMDAR encephalitis OPN: ↑ in anti-NMDAR encephalitis	★★	(25, 51, 76, 79, 80, 82, 89–91)
Nerve damage and glial activity markers	S100 protein	S100 protein include 2 important members, S100A and S100B. S100A can help B lymphocytes to pass through the BBB in AE patients, while S100B is a CNS-specific protein and related to brain injury.	CNS concentration of S100B is closely linked to treatment response, disease severity and prognosis. The level of CSF-Nfl is closely related to prognosis of both anti-NMDAR and anti-LGl1 encephalitis. Patient has either of these 2 types of AE accompanied by CSF-Nfl elevation manifested poor diagnosis and prognosis. The level of Nfl is also closely related to disease severity.	S100 protein: ↑ in anti-NMDAR and anti-DPPX encephalitis PGRN: ↑ in anti-NMDAR encephalitis Nfl:↑ in anti-NMDAR and anti-LGI1 encephalitis GFAP: ↑ in anti-NMDAR and limbic encephalitis Total-tau: ↑ in anti-NMDAR encephalitis	★	(103, 105–108)
	PGRN	PGRN is a multifunctional immunomodulatory molecule which is critical in autoimmune diseases, elevated level of PGRN can be seen in the CSF of patients with anti-NMDAR encephalitis.			★★	(113, 114)
	Nfl	Neurofilament light chain (NfL) are scaffolding proteins expressing specifically on the neural skeleton. They have been used as unspecific markers of axonal damage neuroinflammation. As neuroinflammation usually involves in AE development, the potential linkage between AE and Nfl has been explored.			★★	(114, 115, 118)
	GFAP	GFAP is a key component during astrocyte development, during astrocytes injury, GFAP may enter to the CSF and eventually to the peripheral blood through venous drainage. Astrocytes injury also participates in AE development.	The level of CSF-GFAP is directly related to the final outcome (disability at 1 year) since AE onset.		★★	(101)
	Total-tau	Total-tau is a representative marker of neuronal and axonal loss, which is closely linked to AE.	The level of total-tau is associated with AE severity, patients developing hippocampal sclerosis are prone to have higher level of total-tau. The level of total-tau also link to the disease final outcome.		★	(101, 114)
OCBs	/	OCBs are clones of immune-globulins, their presence can be mediated by autoimmune antibodies which correlated to AE.	CSF OCB positivity rate is included in the clinical diagnostic criteria for both anti-NMDAR encephalitis and possible AE with negative antibodies.	OCBs:↑ in anti-NMDAR anti-GABABR, anti-AMPAR and anti-DPPX encephalitis	★★★	(5, 6, 123, 124, 129)
Markers of synaptic dysfunction	Neurogranin and SNAP-25	Neurogranin and SNAP-25 are both presentative synaptic proteins. While synaptic dysfunction participate in AE development, relative biomarkers may reflect disease	Both neurogranin and SNAP-25 were markedly decreased in the CSF of AME patients at presentation. Lower SNAP-25 in prospectively followed patients and higher neurogranin at presentation is associated with greater disease severity.	Neurogranin: ↑ in anti-NMDAR encephalitis SNAP-25:↑ in anti-NMDAR encephalitis	★★	(135)

## Author Contributions

Drafting/revision of the manuscript for content and including medical writing for content: SZ, XL, and CM. Drafting/revision of the manuscript for content, including medical writing for content, and article frame design: JT and WM. All authors contributed to the article and approved the submitted version.

## Funding

The author(s) disclosed the receipt of the following financial support for the research, authorship, and/or publication of this article: This study was supported by the National Natural Science Foundation of China (Grant 81901300 to CM), the Scientific and Technological Project of Henan Province (Grant SBGJ202003020 to CM), and the Youth Innovation Fund of the First Affiliated Hospital of Zhengzhou University (to SZ).

## Conflict of Interest

The authors declare that the research was conducted in the absence of any commercial or financial relationships that could be construed as a potential conflict of interest.

## Publisher's Note

All claims expressed in this article are solely those of the authors and do not necessarily represent those of their affiliated organizations, or those of the publisher, the editors and the reviewers. Any product that may be evaluated in this article, or claim that may be made by its manufacturer, is not guaranteed or endorsed by the publisher.
